# Pharmacophore modeling: advances and pitfalls

**DOI:** 10.3389/fmolb.2025.1760982

**Published:** 2026-01-07

**Authors:** Mahmoud Y. Elsaka, M. Modather Taha, Amr Tayel, Haytham O. Tawfik, Mahmoud A. A. Ibrahim, Tamer Shoeib

**Affiliations:** 1 Department of Pharmaceutical Chemistry, Faculty of Pharmacy, Alsalam University in Egypt, Tanta, Egypt; 2 Department of Chemistry, The American University in Cairo, New Cairo, Egypt; 3 Department of Pharmaceutical Chemistry, Faculty of Pharmacy, Tanta University, Tanta, Egypt; 4 Computational Chemistry Laboratory, Chemistry Department, Faculty of Science, Minia University, Minia, Egypt; 5 School of Health Sciences, University of KwaZulu-Natal, Westville Campus, Durban, South Africa; 6 Department of Engineering, College of Engineering and Technology, University of Technology and Applied Sciences, Nizwa, Oman

**Keywords:** dynophore, machine learning, multi-pharmacophore, pharmacophore, pitfalls

## Abstract

Pharmacophore modeling has evolved from a static conceptual framework into a central computational tool in modern drug discovery. Recent advances include multi-pharmacophore strategies that better capture ligand diversity and target flexibility, as well as dynamic pharmacophore models (“dynophores”) derived from molecular dynamics simulations that reflect time-dependent interaction patterns. The integration of artificial intelligence and machine learning has further improved feature extraction, virtual screening accuracy, and predictive performance across discovery pipelines. Despite these advances, pharmacophore modeling remains constrained by conformational bias, limited binding-mode representation, and computational cost. Case studies involving efflux pumps, topoisomerase IIα, and LEDGF/p75–integrase inhibitors illustrate both the strengths and limitations of current methods. Collectively, these developments underscore the value of hybrid approaches to enhance pharmacophore reliability and real-world utility.

## 1 Introduction

The concept of pharmacophores represents one of the oldest and most fundamental ideas in medicinal chemistry. The term was first introduced by Paul Ehrlich in the early 20th century to describe the ensemble of steric and electronic features necessary for optimal supramolecular interactions with a specific biological target. Over time, the pharmacophore evolved from a simple theoretical idea into a computational model capable of explaining the essential chemical features responsible for a molecule’s biological activity. With the development of computational chemistry and molecular modeling tools in the 1980s and 1990s, pharmacophore modeling became a practical approach for identifying active compounds, even in the absence of a known receptor structure (Seidel et al., 2019).

Pharmacophore models can generally be categorized into two main types: ligand-based pharmacophores (LBP) and structure-based pharmacophores (SBP). Ligand-based models are generated when the three-dimensional structure of the biological target is unknown; they rely on a set of active compounds to extract shared chemical features such as hydrogen bond donors, acceptors, hydrophobic regions, and aromatic centers. In contrast, structure-based pharmacophores are derived directly from the 3D structure of the target protein–ligand complex, typically obtained through X-ray crystallography or molecular docking. Hybrid approaches have also emerged, combining information from both ligands and receptor structures to enhance model reliability and predictive performance (Sanders et al., 2012).

Pharmacophore modeling provides a simplified yet highly informative representation of the interaction pattern between ligands and their targets. This abstraction allows researchers to identify the “molecular key” that fits a given “biological lock.” Unlike conventional docking, pharmacophore modeling emphasizes the functional arrangement of features rather than the entire molecular structure. This makes it an invaluable approach for hit identification, lead optimization, and scaffold hopping. Moreover, it allows screening of large chemical libraries efficiently by focusing on the essential features responsible for activity, significantly reducing computational cost and increasing the probability of identifying novel active compounds with diverse scaffolds (Seidel et al., 2020).

Pharmacophore-based virtual screening has become a cornerstone of modern drug discovery. Its success lies in its ability to capture the pharmacological essence of active compounds and apply it to the exploration of massive chemical databases. Numerous drugs and lead compounds have been identified using pharmacophore models as the primary screening tool. For instance, pharmacophore-guided approaches have contributed to the discovery of inhibitors targeting kinases, GPCRs, and proteases, among others. The integration of pharmacophore models with molecular docking, molecular dynamics simulations, and quantitative structure–activity relationship (QSAR) studies has further improved their accuracy and predictive power. Recent advances in software such as LigandScout®, MOE®, and Discovery Studio® have automated pharmacophore generation and validation, enabling researchers to design highly specific and multi-target compounds. This capability is especially valuable in the era of polypharmacology, where targeting multiple receptors simultaneously offers promising therapeutic advantages (Pal et al., 2019; Wolber and Langer, 2005).

## 2 Advances in pharmacophore modeling

Over the past decade, pharmacophore modeling has evolved from a largely static and interpretive framework into a dynamic, adaptive, and data-driven model within computer-aided drug design (CADD). The transformation has been driven by improved understanding of molecular flexibility, the integration of large-scale biological and chemical databases, and the rapid advancement of artificial intelligence (AI) and machine learning (ML) methodologies. Modern pharmacophore models are no longer constrained by single hypotheses or rigid description of ligand–receptor interactions. Instead, pharmacophore models incorporate multiple hypotheses, include molecular dynamics (MD) simulations to account for temporal and conformational fluctuations, and utilize AI-based algorithms to automate model generation, refinement, and prediction. Collectively, these methodological developments have significantly reshaped the design and optimization of bioactive compounds by enhancing hit enrichment, improving interpretability, and accelerating the overall discovery process (Steindl et al., 2006; Janežič et al., 2021).

### 2.1 Multiple pharmacophores-based modeling approach

Traditional pharmacophore modeling relies on a single hypothesis describing the key molecular features required for activity, a strategy that performs adequately for rigid targets but poorly for flexible proteins or multi-subpocket systems where ligands adopt diverse conformations and interaction patterns. To address these limitations, multi-pharmacophore approaches have emerged, integrating complementary hypotheses derived from various ligand classes, receptor conformations, or docking poses. This methodology offers a more comprehensive and accurate representation of molecular recognition (Steindl et al., 2006).

A representative example is provided by studies on the *Neisseria gonorrhoeae* MtrD efflux transporter, which effectively illustrate multi-pharmacophore methodology. In this work, researchers developed five pharmacophore models; three ligand-based (derived from PaβN, MBX2319, and D13-9001 analogs) and two structure-based (generated from erythromycin and ampicillin). The erythromycin model featured one hydrogen-bond acceptor and four hydrophobic elements, whereas the ampicillin model comprised one hydrogen-bond acceptor, two hydrophobic moieties, and an aromatic ring enabling π–π interactions with key phenylalanine residues. Although both hypotheses described the same binding site, they captured distinct yet complementary interaction patterns reflective of different chemical perspectives (Jain et al., 2022).

Using the ZINC natural products database as the screening source, the pharmacophore models produced markedly different initial hit counts: 556 for PaβN, 1,707 for D13-9001, 23,520 for MBX2319, 15,824 for erythromycin, and 12,601 for ampicillin. Following multistage refinement through HTVS, SP, and XP docking, fewer than 1% of candidates were retained. The wide variation in retention ratios (0.2%–12%) suggested that broader pharmacophores capture a wider range of scaffolds but also generate more false positives, whereas more selective models identify fewer yet higher-quality binders. This analysis highlights the principal advantage of the multi-pharmacophore strategy: broad hypotheses enhance chemical diversity, and restrictive hypotheses provide precision. Their combined application yields a balanced screening framework capable of uncovering novel bioactive scaffolds (Jain et al., 2022).

The multi-pharmacophore modeling strategy is particularly powerful in its ability to integrate multiple complementary hypotheses, thereby uniting chemical diversity with structural rigor. This integration enhances hit reliability, minimizes bias arising from any single ligand or receptor conformation, and yields a more realistic representation of ligand–target recognition—an advantage especially relevant to highly flexible systems such as efflux pumps and GPCRs. However, the approach is not without limitations. Its computational demands are substantial, and the generation of overlapping or competing hits can complicate downstream analysis. Furthermore, because pharmacophore models are inherently static constructs, they capture protein dynamics only superficially and require molecular dynamics simulations to adequately account for conformational flexibility (Schönherr et al., 2024).

### 2.2 Dynamic pharmacophores: integration with molecular dynamics simulation

Dynamic pharmacophore modeling, often referred to as the “dynophore” approach, extends classical pharmacophore design by incorporating the temporal dimension of protein–ligand interactions obtained from molecular dynamics (MD) simulations. Unlike static models derived from a single conformational state, dynophores capture time-averaged hydrogen-bonding patterns, hydrophobic contacts, and conformational fluctuations throughout the trajectory, thereby offering a more realistic and comprehensive representation of molecular recognition processes (Mortier et al., 2015; Hayek-Orduz et al., 2025; Janežič et al., 2021).

**FIGURE 1:**
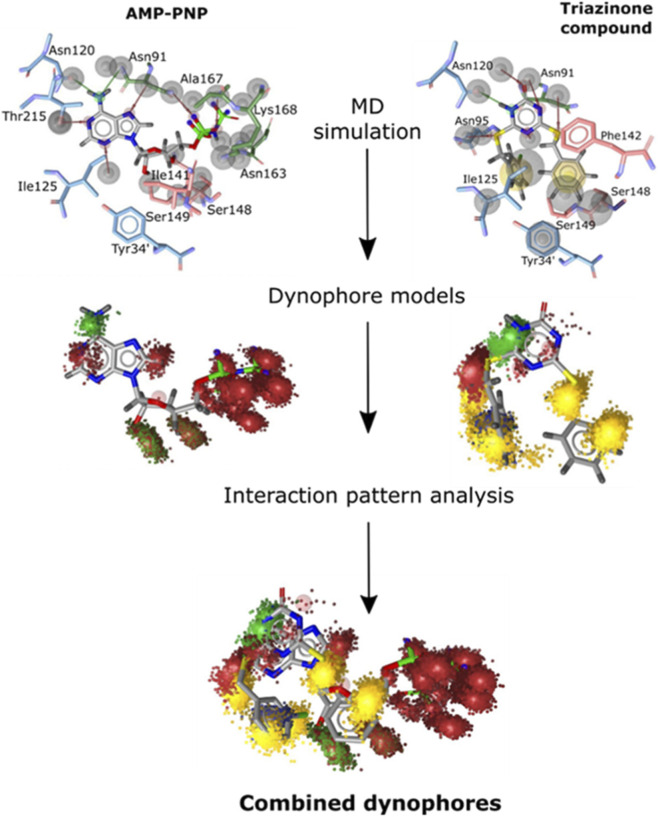
Dynophore generation from MD simulations of AMP-PNP and a triazinone inhibitor, followed by analysis and integration of the resulting dynamic pharmacophore models (reprinted from Janežič et al., 2021, licensed under CC BY 4.0).

A representative application of this strategy is demonstrated in the identification of novel inhibitors of human DNA topoisomerase IIα, as shown in Figure 1. Molecular dynamics simulations were conducted for both the native ATP analog (AMP-PNP) and a triazinone-based catalytic inhibitor within the ATP-binding pocket. Interaction profiles extracted from these simulations were used to generate two individual dynophore models, which were subsequently integrated into a single composite pharmacophore capturing persistent hydrogen bonds such as those with Asn120, and stable hydrophobic contacts with residues including Ile125 and Phe142. Three derived submodels were then applied to virtually screen approximately 32,000 natural products, yielding flavonoid candidates, most notably epicatechin gallate, with potent catalytic inhibition (IC_50_ = 1.7 µM). Follow-up biochemical assays and MD analyses confirmed selective binding to the ATPase domain, thereby validating the predictive capability of the dynophore-based design methodolgy (Janežič et al., 2021).

**FIGURE 2:**
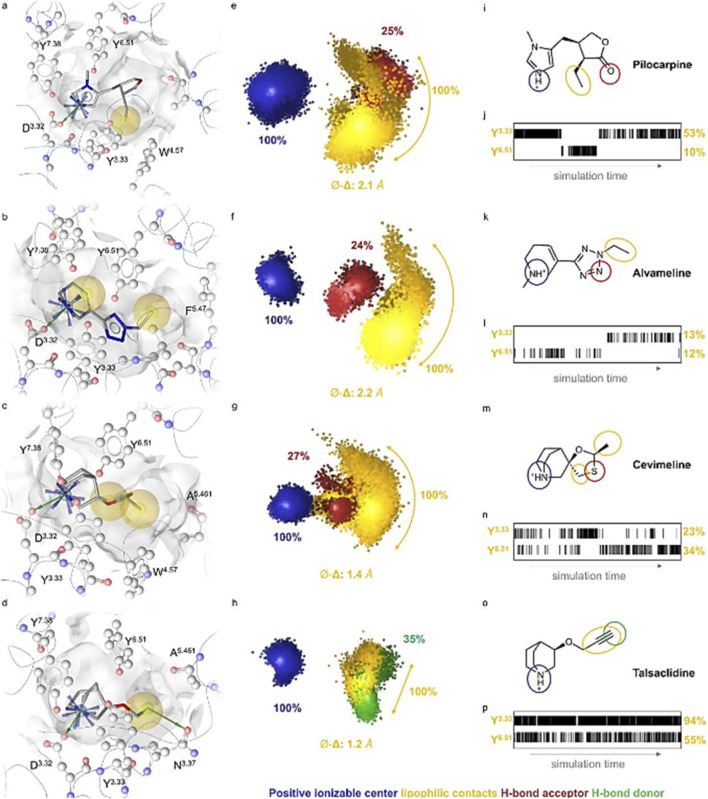
Static pharmacophores **(a–d)**, dynophores **(e–h)**, corresponding 2D ligand structures **(i, k, m, o)**, and residue contact barcodes **(j, l, n, p)** for pilocarpine, alvameline, cevimeline, and talsaclidine in the M_2_ receptor orthosteric site derived from triplicate molecular dynamic simulations (reprinted from Wunsch et al., 2025, licensed under CC-BY-NC-ND 4.0).

Another representative application of dynamic pharmacophore modeling of GPCR as shown in Figure 2 was reported by Wunsch et al., who employed dynophore analysis to investigate partial agonism at the muscarinic M_2_ receptor. Despite sharing a conserved positively ionizable anchoring interaction within the orthosteric site, classical partial agonists, including pilocarpine, alvameline, cevimeline, and talsaclidine, exhibited distinct, time-dependent binding behaviors in molecular dynamics simulations. Dynophore models derived from these trajectories captured differences in the spatial distribution of lipophilic features, providing a dynamic description of ligand–receptor interactions consistent with partial agonist binding modes (Wunsch et al., 2025).

Notably, differences in the spatial dispersion of lipophilic interaction features were associated with experimentally measured agonist efficacies. Lower-efficacy ligands, such as pilocarpine, exhibited more diffuse and heterogeneous dynophore patterns, whereas higher-efficacy agonists, including cevimeline and talsaclidine, showed more compact and conformationally restricted interaction profiles. Dynophore analysis further revealed time-dependent switching between interactions with key residues Y^3·^ ^33^ and Y^6·^ ^51^, capturing dynamic binding mode interconversions that are not accessible using static pharmacophore models. The functional relevance of these dynamic interaction signatures was supported experimentally, as the negative allosteric modulator alcuronium selectively attenuated pilocarpine efficacy in a manner consistent with dynophore-predicted stabilization of inactive receptor conformations (Wunsch et al., 2025).

The dynophore approach captures protein flexibility and the time-dependent evolution of intermolecular interactions, thereby offering a more realistic representation of molecular recognition and improving the reliability of virtual screening outcomes. By aligning computational predictions more closely with experimental behavior, it strengthens the validity of structure-based drug design. Nonetheless, the approach is computationally demanding due to its reliance on extensive molecular dynamics simulations and the need for expert analysis. Moreover, limited simulation times may fail to sample slower or large-scale conformational transitions, potentially restricting its capacity to represent long-range protein dynamics (Janežič et al., 2021).

### 2.3 AI-integrated pharmacophore modeling

Artificial intelligence (AI) has emerged as a transformative driver in modern drug discovery, especially when integrated with established computational frameworks such as pharmacophore modeling. This synergy enables the convergence of large-scale, data-driven inference with mechanistic chemical insight, thereby improving both target characterization and the precision of virtual screening pipelines. AI techniques can systematically interrogate extensive structural, biochemical, and omics datasets to identify previously unrecognized binding pockets, infer ligandability, and prioritize molecular features associated with biological activity. Pharmacophore modeling subsequently translates these predictions into interpretable three-dimensional interaction maps that capture essential hydrogen-bonding, electrostatic, and hydrophobic features, providing a rational foundation for ligand design and optimization. Collectively, the integration of AI with pharmacophore-based methods strengthens predictive performance, enhances model interpretability, and accelerates the discovery of novel chemical scaffolds (Paul et al., 2021).

Recent progress in AI-driven pharmacophore modeling has led to the development of PharmacoMatch, an embedding-based framework that jointly encodes pharmacophoric feature types and their three-dimensional spatial arrangements. By representing pharmacophores as vectors in a learned latent space, the method enables fast and scalable comparison of pharmacophoric patterns while preserving sensitivity to feature identity and position. Dimensionality-reduction analyses demonstrate that pharmacophores with similar characteristics cluster closely, and controlled perturbation studies confirm that small spatial displacements produce measurable declines in matching scores, indicating effective incorporation of 3D information (Rose et al., 2024). PharmacoNet, in contrast, employs deep learning primarily for pharmacophore generation and binding pose estimation, rather than direct vector-based matching (Seo and Kim, 2024).

While PharmacoNet requires alignment-based computations to compare pharmacophores, PharmacoMatch encodes pharmacophoric patterns into a latent space, allowing rapid similarity searches without explicit alignment. This vector-based approach preserves 3D positional and feature-type information, resulting in significantly faster screening with comparable or improved enrichment performance (Rose et al., 2024). In a large-scale virtual screening application, PharmacoMatch was evaluated on multiple targets from the DUD-E benchmark (Mysinger et al., 2012), achieving enrichment performance comparable to classical alignment-based methods such as CDPKit (Seidel, 2025), but with substantially reduced computational cost and no manual query refinement. Further benchmarking against PharmacoNet (Seo and Kim, 2024) on the DEKOIS2.0 (Bauer et al., 2013) and LIT-PCBA (Nguyen et al., 2020) datasets showed improved early enrichment metrics and orders-of-magnitude faster runtimes, as reported by (Rose et al., 2024).

Collectively, these examples demonstrate how AI-integrated pharmacophore representations has become a functional and increasingly indispensable component of modern drug discovery, enhancing predictive accuracy, enabling rapid hypothesis generation, and supporting more efficient progression from early design to clinical evaluation, while highlighting remaining challenges related to fine-grained geometric discrimination.

## 3 Pitfalls and limitations in pharmacophore modeling

### 3.1 Conformational restrictions that come up with pharmacophores

Pharmacophores, as discussed earlier, are sets of three-dimensional features that capture the essential functional characteristics of chemical compounds and the spatial relationships between these features. They provide a powerful framework for identifying novel hit compounds against specific biological targets, making pharmacophore modeling a widely used technique in virtual screening. Despite its utility, this approach is limited by conformational restrictions inherent to the algorithms used for pharmacophore generation, a challenge that affects both ligand-based and structure-based models (Cao, 2025).

In ligand-based pharmacophore modeling, the approach begins with a set of known active ligands, from which the common spatial features are extracted to create a pharmacophoric hypothesis. The assumption is that any compound capable of matching these features will adopt the bioactive conformation within the target protein and thus exhibit biological activity. While this seems logical, it introduces a fundamental pitfall. In reality, active compounds are not static; they exist as dynamic ensembles of interconverting conformations. The bioactive conformation represents only one of these possibilities, which may not be adequately sampled or represented during model generation or virtual screening (Muhammed and Aki-Yalcin, 2021).

Consequently, a compound that aligns with the pharmacophore does not necessarily adopt the correct binding mode experimentally; it simply contains the spatial features shared by the training ligand set. In this sense, ligand-based pharmacophores are inherently restricted to fixed conformations, and hits that appear compatible with the pharmacophore may ultimately fail to interact productively with the target (Ghosh et al., 2025). The latter describes a limitation accompanied with ligand based models from the conformation perspective, although conformer generation algorithms utilized in ligand based pharmacophore modeling can be successful (Sanders et al., 2012; Rampogu, 2025). Structure-based pharmacophore modeling offers a partial solution to this limitation by using the bioactive conformation of a co-crystallized ligand as a template. This provides a reliable reference for one possible binding mode within the target pocket. However, even here, the challenge remains. A hit identified through a structure-based pharmacophore may theoretically satisfy the spatial feature requirements, yet it may not adopt the same binding mode experimentally (Giordano et al., 2022).

Differences in molecular structure, flexibility, and energetic preferences mean that the compound may require an alternative conformation to bind effectively. If it does adopt a different conformation, the intended interactions may not occur, and the binding may depend largely on chance rather than predictive accuracy (McNutt et al., 2023; Janežič et al., 2021). Overall, both ligand-based and structure-based pharmacophore models offer valuable insights for hit identification, yet they are constrained by assumptions about molecular conformation and feature alignment. While these methods remain essential tools in virtual screening, they cannot fully guarantee that a predicted hit will engage the target protein as intended. The challenge of conformational oversimplification continues to be a central limitation, underscoring the importance of integrating complementary approaches that account for molecular flexibility and the dynamic nature of protein-ligand interactions (Sanders et al., 2012; McNutt et al., 2023).

### 3.2 A bias and chance: pharmacophore modeling from different perspectives

Pharmacophore modeling fundamentally functions as a classification technique, categorizing molecules in a screening database as potential hits or non-hits. Like any classification model, it is inherently prone to bias, which can significantly impact its predictive performance. In ligand-based pharmacophore modeling, such biases are often observed in the form of conformational and scaffold biases, especially when the model is trained on a set of structurally similar compounds (Sousa et al., 2023). In contrast, structure-based pharmacophore modeling is prone to a different type of bias, commonly referred to as limited interaction bias, which arises from the restricted set of receptor-ligand interactions captured during model construction (Sousa et al., 2023).

By relying on the common pharmacophoric features derived from such a limited or homogeneous set, the resulting model may overemphasize a specific scaffold or binding mode. This creates a bias toward a narrow region of the chemical space, under the assumption that a highly selective pharmacophore will capture all active compounds. In reality, proteins often accommodate multiple interaction patterns or alternative binding modes, meaning that a model biased toward a single conformational or scaffold pattern may fail to identify other valid hits (Ajjarapu et al., 2022). Attempting to correct this bias by generalizing the model, for example, by increasing the number of active compounds in the training set, can reduce the model’s classification power. This is often reflected in a decrease in the enrichment factor, highlighting a trade-off between broader chemical coverage and predictive selectivity. One practical approach to overcome conformational or scaffold bias is to employ multiple ligand-based pharmacophore models, each derived from different sets of actives, thereby capturing a wider range of potential interaction patterns without overly constraining the model to a single scaffold (Shah and Jain, 2022).

Structure-based pharmacophore models, while partially addressing the limitations of ligand-based approaches, are also susceptible to bias. Conformational restrictions inherent in the model can lead to binding mode bias, as the pharmacophore is constructed based on a specific co-crystallized ligand. However, many ligands may interact with the protein in alternative binding modes, which the model may not fully capture. Furthermore, chance continues to play a significant role in pharmacophore modeling (Kumar et al., 2022). The translation of predictions from computational models to actual experimental outcomes remains imperfect. Biological systems are dynamic and complex, whereas pharmacophore algorithms rely on static representations of molecular interactions. This disconnect explains why many top-ranking hits identified by highly selective ligand- or structure-based pharmacophores fail in biological assays. In such cases, experimental success may sometimes result more from chance than from the inherent discriminative power of the pharmacophore model (Braga and Andrade, 2013; Kumar et al., 2022).

### 3.3 Failures of pharmacophore modeling

Pharmacophore model limitations can be found in efforts to develop inhibitors of the LEDGF/p75–integrase interaction, a key target in HIV therapeutics. Even when pharmacophore models are generated from similar datasets, they can capture slightly different features, leading to distinct hit molecules in virtual screening. This illustrates how the subjective choices made during model construction can produce inconsistent results, potentially causing promising leads to be overlooked and slowing down the drug development process (Yang, 2010).

A similar situation arises with cyclin-dependent kinase 2 (CDK2) inhibitors, where ligand-based pharmacophore models often vary in the types of features and their spatial arrangements, depending on the training set used. These examples highlight the inherent limitations of conventional pharmacophore modeling and underscore the value of hybrid strategies, such as incorporating molecular dynamics simulations or machine learning, to improve predictive accuracy. Overall, while pharmacophore modeling is a powerful tool for accelerating early-stage drug discovery, these cases remind us that careful validation and the use of complementary methods are essential to ensure reliable results and reduce the risk of failure in pharmaceutical research (Qing et al., 2014).
